# Survival of pathogenic and lactobacilli species of fermented olives during simulated human digestion

**DOI:** 10.3389/fmicb.2014.00540

**Published:** 2014-10-14

**Authors:** Francisco N. Arroyo-López, Stéphanie Blanquet-Diot, Sylvain Denis, Jonathan Thévenot, Sandrine Chalancon, Monique Alric, Francisco Rodríguez-Gómez, Verónica Romero-Gil, Rufino Jiménez-Díaz, Antonio Garrido-Fernández

**Affiliations:** ^1^Biotecnología de Alimentos, Instituto de la Grasa – Consejo Superior de Investigaciones CientíficasSeville, Spain; ^2^Centre de Recherche en Nutrition Humaine Auvergne, EA 4678, Conception Ingénierie et Développement de l’Aliment et du Médicament, Clermont Université – Université d’AuvergneClermont-Ferrand, France; ^3^Centre de Recherche en Nutrition Humaine Auvergne, M2iSH, UMR INSERM/Université d’Auvergne U1071 USC-INRA 2018, Clermont Université – Université d’AuvergneClermont-Ferrand, France

**Keywords:** food carrier, lactobacilli, pathogen, probiotic, survival, Shiga toxins, table olives, TIM system

## Abstract

The present survey uses a dynamic gastric and small intestinal model to assess the survival of one pathogenic (*Escherichia coli* O157:H7 EDL 933) and three lactobacilli bacteria with probiotic potential (*Lactobacillus rhamnosus* GG, *L. pentosus* TOMC-LAB2, and *L. pentosus* TOMC-LAB4) during their passage through the human gastrointestinal tract using fermented olives as the food matrix. The data showed that the survival of the *E. coli* strain in the stomach and duodenum was very low, while its transit through the distal parts (jejunum and ileum) resulted in an increase in the pathogen population. The production of Shiga toxins by this enterohemorrhagic microorganism in the ileal eﬄuents of the *in vitro* system was too low to be detected by ELISA assays. On the contrary, the three lactobacilli species assayed showed a considerable resistance to the gastric digestion, but not to the intestinal one, which affected their survival, and was especially evident in the case of both *L. pentosus* strains. In spite of this, high population levels for all assayed microorganisms were recovered at the end of the gastrointestinal passage. The results obtained in the present study show the potential use of table olives as a vehicle of beneficial microorganisms to the human body, as well as the need for good hygienic practices on the part of olive manufacturers in order to avoid the possibility of contamination by food-borne pathogens.

## INTRODUCTION

Green Spanish-style table olives are considered ready-to-eat products which are directly consumed without any prior cooking, and often, without any pasteurization or sterilization treatment. This makes olives a splendid vehicle of microorganisms (beneficial or harmful) to the human body. Thereby, the analysis of the microbiota adhered to the fruits acquires a relevant importance, although, incomprehensibly, this point has received scarce attention by researchers. Fermented olives contain biofilm structures formed mainly by lactic acid bacteria (LAB) and yeasts, which can reach population levels up to 8 log_10_ CFU g^-1^ in the epidermis of the fruits (Nychas et al., 2002; Arroyo-López et al., 2012; Domínguez-Manzano et al., 2012). Native LAB or yeast strains isolated from olive microbiota which show potential probiotic characteristics could be used as starters either to initiate fermentation or to obtain a functional product after adhesion to the fruits (Rodríguez-Gómez et al., 2013; Blana et al., 2014). To exert their health effect, microorganisms to be evaluated as probiotics should be able to survive the harsh conditions found in the human digestive tract such as acidic pH or high concentrations of digestive enzymes and bile salts.

Regarding safety issues, it is necessary to provide more knowledge about the behavior of pathogens in ready-to-eat fermented olives for future risk assessments. Enterohemorrhagic *Escherichia coli* is a major food-borne pathogen that causes hemorrhagic colitis and a life-threatening sequelae, the hemolytic uremic syndrome (Pennington, 2010). The behavior of the enterohemorrhagic *E. coli* O157:H7 during olive processing (fermentation and packing) has been reported by several authors (Spyropoulou et al., 2001; Skandamis and Nychas, 2003; Argyri et al., 2013; Grounda et al., 2013) but there is hitherto no available information about the survival of this pathogen in the case of a hypothetical intake of contaminated olives. To cause human illness, *E. coli* O157:H7 should not only survive its passage through the gastrointestinal tract but also coordinate the expression of virulence genes especially that of encoding Shiga toxins (Foster, 2013).

Most of the available data on probiotic or pathogen survival in the human digestive tract has been obtained in static *in vitro* systems that are not representative of the continuously changing variable during the gastrointestinal transit. The TNO gastrointestinal tract model (TIM system, Zeist, Netherlands) is an alternative dynamic multi-compartmental *in vitro* system which currently allows the closest simulation of *in vivo* physiological processes occurring within the stomach and small intestine of humans (Minekus et al., 1995; Guerra et al., 2012).

In this work, we used the TIM system to assess the survival and toxin production of an enterohemorrhagic *E. coli* strain inoculated in fermented olives during its transit through the upper human gastrointestinal tract. The survival of two potential probiotic strains isolated from table olives and belonging to *Lactobacillus pentosus* species (never studied before in dynamic digestive assays) was also evaluated and compared to that of a well-recognized probiotic microorganism (*L. rhamnosus* GG).

## MATERIALS AND METHODS

### OLIVES AND MICROORGANISMS

Fermented olives of the *Manzanilla* variety (*Olea europaea pomiformis*) were used in the present study. The fruits were previously fermented for two months according to the Spanish-style (Garrido-Fernández et al., 1997) and then pasteurized at 80°C for 15 min to avoid any microbial presence on the olive surface. Twenty-five grams of pasteurized olives were then homogenized with 250 mL of sterile water and independently inoculated with aerobic cultures of the reference strain *E. coli* O157:H7 EDL 933 (LB, 24 h, 37°C), the probiotic *L. rhamnosus* GG, and the olive isolates *L. pentosus* TOMC-LAB2 and *L. pentosus* TOMC-LAB4 (MRS broth, 24 h, 37°C) to reach a final population in the mix of 8.2, 9.1, 9.7, and 9.4 log_10_ CFU, respectively. Except for the pathogen strain, these inoculation levels are similar for the lactic acid population usually present in 25 g of fermented olives. The two *L. pentosus* strains, originally isolated from Spanish-style table olive fermentations and belonging to the table olive microorganisms collection (TOMC) from Food Biotechnology Department of Instituto de la Grasa (CSIC-Seville), were selected because of their previously described probiotic characteristics (Bautista-Gallego et al., 2013) and good performance as starter in previous fermentation trials carried out at laboratory scale (Rodríguez-Gómez et al., 2013). **Table 1** shows the main physicochemical conditions of the fermented olives at the moment of introduction into the TIM system.

**Table 1:** Experimental design and physicochemical conditions of the fermented olives used in the present work as food matrix.

Reference for treatment	Inoculated microorganism	Attributed effects on human health	Physicochemical conditions of fermented olives*
Ec	*Escherichia coli* O157:H7 EDL 933	Enterohemorrhagic pathogen	pH = 4.1 Salt = 50.0 g/l
Lr	*Lactobacillus rhamnosus* GG	Probiotic microorganism	Sugars ≤ 2.0 g/l
Lp2	*L. pentosus* LAB2	Potential probiotic strains	Phenolic compounds = 900 mg/kg
Lp4	*L. pentosus* LAB4	Isolated from table olives	

*Fruits had an average size of 4.06 ± 0.49 g and area surface of 10.93 ± 1.07 cm^2^.

### SIMULATED HUMAN DIGESTIVE CONDITIONS

The TIM system, which simulates the physiological processes occurring in the stomach, duodenum, jejunum, and ileum, was programmed to reproduce the digestion of a solid food matrix in a healthy human adult using the protocol described by Etienne-Mesmin et al. (2011). The total duration of the digestions was 300 min with *n = 2* digestions for each different microorganism. The parameters used for *in vitro* digestion are described in **Table 2**. Samples were taken in the test meal (initial intake) after inoculation and before its introduction into the artificial stomach, and regularly collected during digestion in the different compartments of the system (stomach, duodenum, jejunum, and ileum) as well as in the cumulative ileal eﬄuents (CIE). Microbial counting was performed on LB agar (for *E. coli*) or on MRS agar (for the lactobacilli strains; Oxoid LTD, Basingstoke, Hampshire, England). Because food and microorganisms are in continuous transit from one compartment to the next, in order to assess microbial survival rates in the TIM system, control digestions (*n = 2*) were carried out with the same protocol conditions used for microorganisms, but with water containing only 0.8% (w/v) of blue dextran (Minekus et al., 1995). This compound is a non absorbable transit marker, which will represent a 100% survival percentage for bacteria. Thereby, curves below the transit marker will represent the mortality of the microorganism, while curves above the transit marker will be indicative of bacteria growth renewal.

**Table 2:** Parameters of gastrointestinal digestion in the TIM system when simulating digestive conditions of a healthy adult after intake of a solid food matrix.

Compartment	Vol (ml)	pH/time (min)	Secretion	t_1/2_ (min)	β coefficient
Stomach	300	2/0, 6/5, 5.7/15, 4.5/45,2.9/90, 2.3/120, 1.8/240, 1.6/300	0.25 ml/min of pepsin (2080 IU/ml), 0.25 ml/min of lipase (250.5 IU/ml), 0.25 ml/min of HCl (1.5 M) if necessary.	85	1.8
Duodenum	30	Maintained at 6.0	0.5 ml/min of bile salts (4% during the first 30 min of digestion and then 2%), 0.25 ml/min of pancreatic juice (10^3^ USP/ml), 0.25 ml/min of intestinal electrolyte solution, 0.25 ml/min of NaHCO_3_ (1 M) if necessary, 23,600 IU of trypsin (at the beginning of digestion).
Jejunum	130	Maintained at 6.9	0.25 ml/min of NaHCO_3_ (1 M) if necessary.		
Ileum	130	Maintained at 7.2	0.25 ml/min of NaHCO_3_ (1 M) if necessary.	250	2.5

*For each digestive compartment, the table gives the volume (initial volume for gastric compartment, maximal volume for intestinal compartments), the pH (depending on the time of digestion in the stomach), and the flow rates of digestive enzymes and bile salts. HCl and NaHCO_3_ are added to the gastric and intestinal compartments, respectively, if the measured value of the pH deviates from the set point. The power exponential equation (*f* = 1–2^-(^*^t^*^/^*^t^*^1/2)β^, where *f* represents the fraction of meal delivered, *t* the time of delivery, *t*_1/2_ half the time of delivery, and β a coefficient describing the shape of the curve) was used for the computer control of gastric and ileal deliveries into the TIM system. Parameters used for t_1/2_ and β for gastric and ileal deliveries are mentioned in the table.*

### DETERMINATION OF THE PRODUCTION OF SHIGA TOXINS BY THE *E. coli* STRAIN

Shiga toxins produced by *E. coli* O157:H7 EDL 933 in the ileal eﬄuents of the TIM system were dosed by enzyme linked immunosorbent assay (ELISA) using the Ridascreen^®^Verotoxin kit (R-Biopharm, Darmstadt, Germany). Purified toxin Stx2 (Toxin Technology^®^, Sarasota, FL, USA) was used to establish standard calibration curves. Samples were diluted to half with the provided diluent and the dosage was made according to the manufacturer’s instructions. Each digestive sample was analyzed in duplicate and serial dilutions of purified toxin were analyzed in triplicate.

### STATISTICAL ANALYSIS

Significant differences among treatments (*p* ≤ 0.05) were tested by analysis of variance (ANOVA) followed by a Fisher-LSD *post hoc* comparison test carried out with STATISTICA 7.0 software package (Statsoft Inc, Tulsa, OK, USA).

## RESULTS AND DISCUSSION

In the present study, fermented olives were independently inoculated with a food-borne pathogen and three potential beneficial microorganisms in order to determine their survival during their passage through the upper gastrointestinal tract of humans and the pathogen’s ability to produce toxins. So far, there is no information available on the survival of microorganisms through the different compartments of the human gastrointestinal tract using table olives as the food matrix. The only related study was carried out by Lavermicocca et al. (2005), who fed volunteers with olives inoculated with the human origin strain *L. paracasei* IMPC2.1. These authors were able to recover the mentioned microorganism from fecal samples, but they did not obtain information on its resistance to specific gastric and intestinal conditions.

**FIGURE 1:**
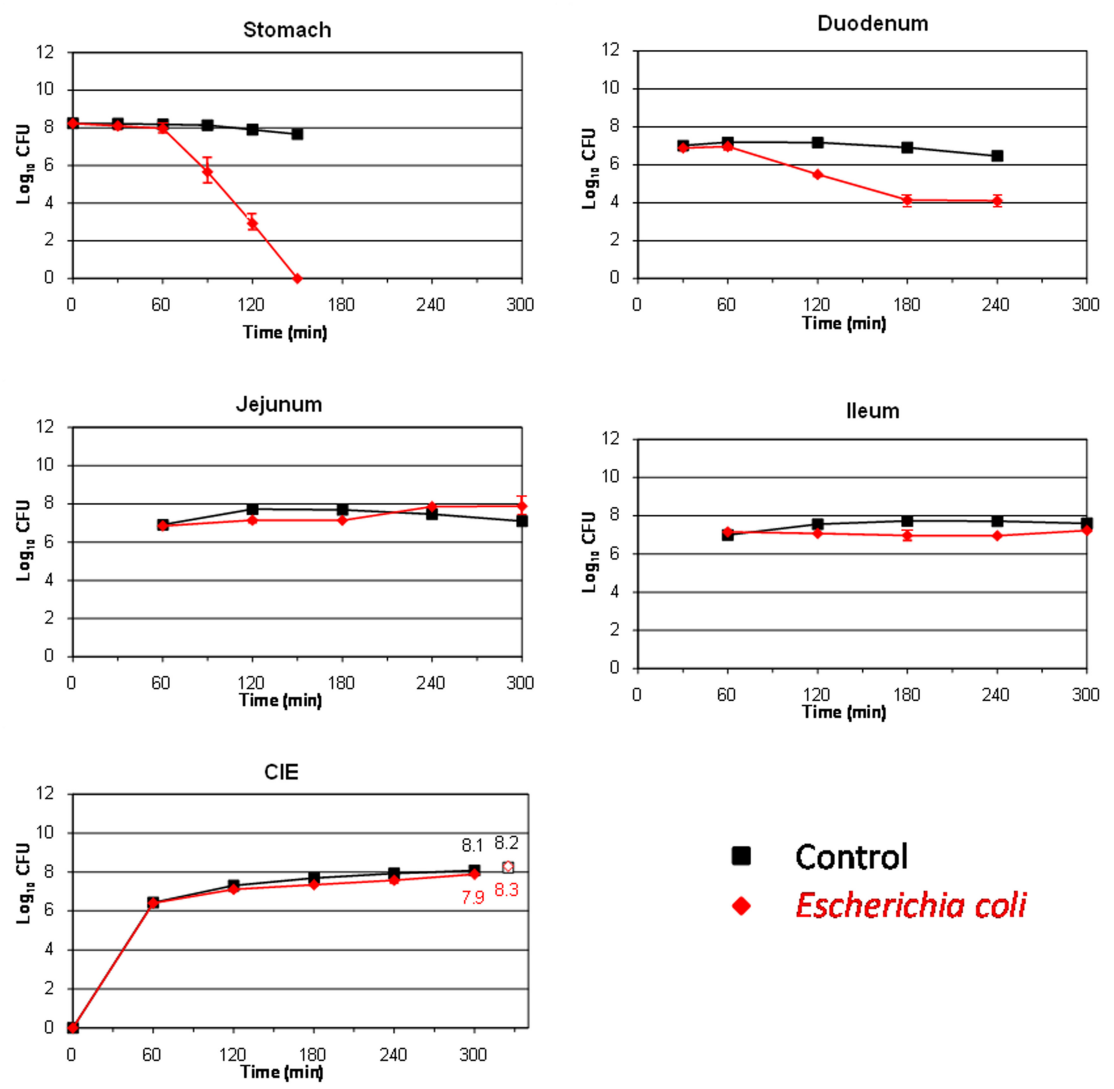
**Survival (plate counts versus time) of the food-borne pathogen *Escherichia coli* O157:H7 EDL 933 in the different compartments and ileal eﬄuents (CIE) of the TIM system compared to a non absorbable transit marker, blue dextran, used as control.** Results are expressed as log_10_ cfu ± SDs (*n* = 2).

The behavior of the major food borne pathogen *E. coli* O157:H7 (strain EDL 933) during its transit through the different compartments of the TIM system is shown in **Figure 1**. In the stomach and duodenum, its population decreased considerably, probably due to the occurrence of stringent conditions such as gastric acidity, digestive enzymes, or bile salts (Smith, 2003). The highest cell mortality was observed in the artificial stomach from 90 min onward, when the pH fell below 3 units (see **Table 2**). At this time, the population decreased by more than 2 log_10_ CFU compared to the transit marker used as control, and no living cells were recovered from this compartment at 150 min. In the duodenum, at 180 min, around 3 log_10_ CFU were lost compared to the marker.

On the contrary, in the distal parts of the artificial gastrointestinal tract (jejunum and ileum), *E. coli* O157:H7 resumption was observed especially at the end of digestion. Thereby, at time 300 min, the counts for bacteria even exceeded that of the transit marker, chiefly in the jejunum (increase of 0.5 log_10_ CFU compared to the control marker). At 300 min, a high amount of viable cells were recovered from the ileal eﬄuents, decreasing only by 0.2 log_10_ CFU with respect to the blue dextran. Bacterial growth renewal has been previously observed in the distal compartments of the TIM system for other *E. coli* strains (Ganzle et al., 1999; Etienne-Mesmin et al., 2011; Miszczycha et al., 2014). This growth resumption was probably linked to less stringent environmental conditions, such as a pH closer to neutrality, lower concentrations of bile salts (owing to their passive reabsorption in the TIM system), and/or an increase in the residence time of bacteria. This event led to an increase in the number of bacteria which could potentially enter into the colon and may potentiate the harmfulness of the food-borne pathogen in the hypothetical case of an intake of contaminated olives.

Large variations in survival rates have been obtained for *E. coli* O157:H7 in diverse *in vitro* digestion assays (Arnold and Kaspar, 1995; Takumi et al., 2000; Foster, 2004; Tamplin, 2005; Etienne-Mesmin et al., 2011). This wide range of response may be explained by differences between culture conditions, digestive systems (static or dynamic) and parameters, food matrices and also bacterial strains. Interestingly, the only studies that evaluated the behavior of *E. coli* O157:H7 in dynamic conditions gave survival rates after gastric digestion close to those obtained in the present work (Takumi et al., 2000; Etienne-Mesmin et al., 2011; Miszczycha et al., 2014). In table olive packing, this microorganism has shown also a considerable survival being present until 19th days of storage on olive fruits at low pH (4.2) and high salt concentration (60 g/l; Argyri et al., 2013).

Hitherto, there is no available data regarding the production (site and amount) of Shiga toxins by enterohemorrhagic *E. coli* strains during their transit through the human gastrointestinal tract. This study is the first to investigate toxin production by this food-borne pathogen in a human simulated digestive environment. Toxin levels in the ileal eﬄuents of the TIM system were too low to be quantified by ELISA assays, which had a detection threshold value of 0.31 ng/ml (data not shown), indicating that under the tested conditions the pathogen was not able to produce Shiga toxins, its main virulence factor, at least at sufficient levels to be detected. It also suggests that toxin production, if it occurs, would rather take place in the colon, which is already described as the main site of pathogenicity for EHEC strains (Shigeno et al., 2002).

**FIGURE 2:**
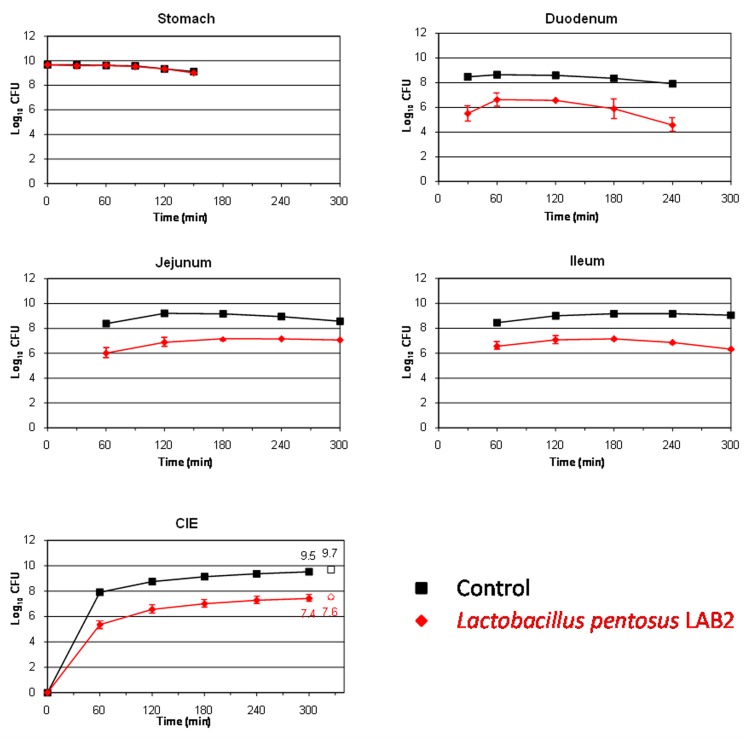
**Survival (plate counts versus time) of the potential probiotic strain *Lactobacillus pentosus* TOMC-LAB2, isolated originally from table olives, in the different CIE of the TIM system compared to a non absorbable transit marker, blue dextran, used as control.** Results are expressed as log_10_ cfu ± SDs (*n* = 2).

**FIGURE 3:**
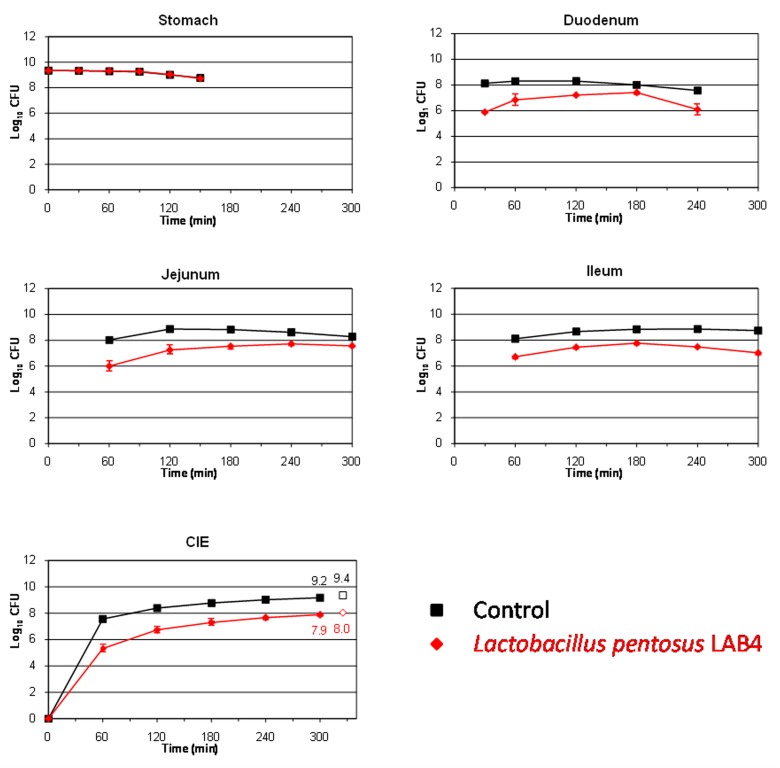
**Survival (plate counts versus time) of the potential probiotic strain *L. pentosus* TOMC-LAB4, isolated originally from table olives, in the different CIE of the TIM system compared to a non absorbable transit marker, blue dextran, used as control.** Results are expressed as log_10_ cfu ± SDs (*n* = 2).

This study also aims to assess the survival of two *L. pentosus* strains isolated from table olives for their use as putative probiotics. LAB species are well known for their ability to resist and live in many different acidified fermented vegetables, among them table olives (Hurtado et al., 2012). The *L. pentosus* strains assayed in this work were originally isolated from table olive fermentations, so, presumably, they should be well adapted to the acidic environment which governs this type of product. **Figures 2** and **3** show the results obtained for the survival in the TIM system of *L. pentosus* TOMC-LAB2 and TOMC-LAB4, respectively. Both bacteria showed a considerable resistance to gastric digestion with a slight decrease at time 150 min of less than 0.1 log_10_ CFU with respect to the transit marker. However, their survival through the duodenum was considerably lower, especially evident in the case of the *L. pentosus* TOMC-LAB2 strain with a reduction of more than 3 log_10_ CFU at 240 min. On the contrary, the survival of neither microorganism was affected by the conditions encountered in the distal parts of the gastrointestinal tract (jejunum and ileum). At 300 min, compared to the transit marker, the amount of bacteria recovered from the ileal eﬄuents was reduced by 2.1 and 1.3 log_10_ CFU for *L. pentosus* TOMC-LAB2 and TOMC-LAB4 strains, respectively.

**FIGURE 4:**
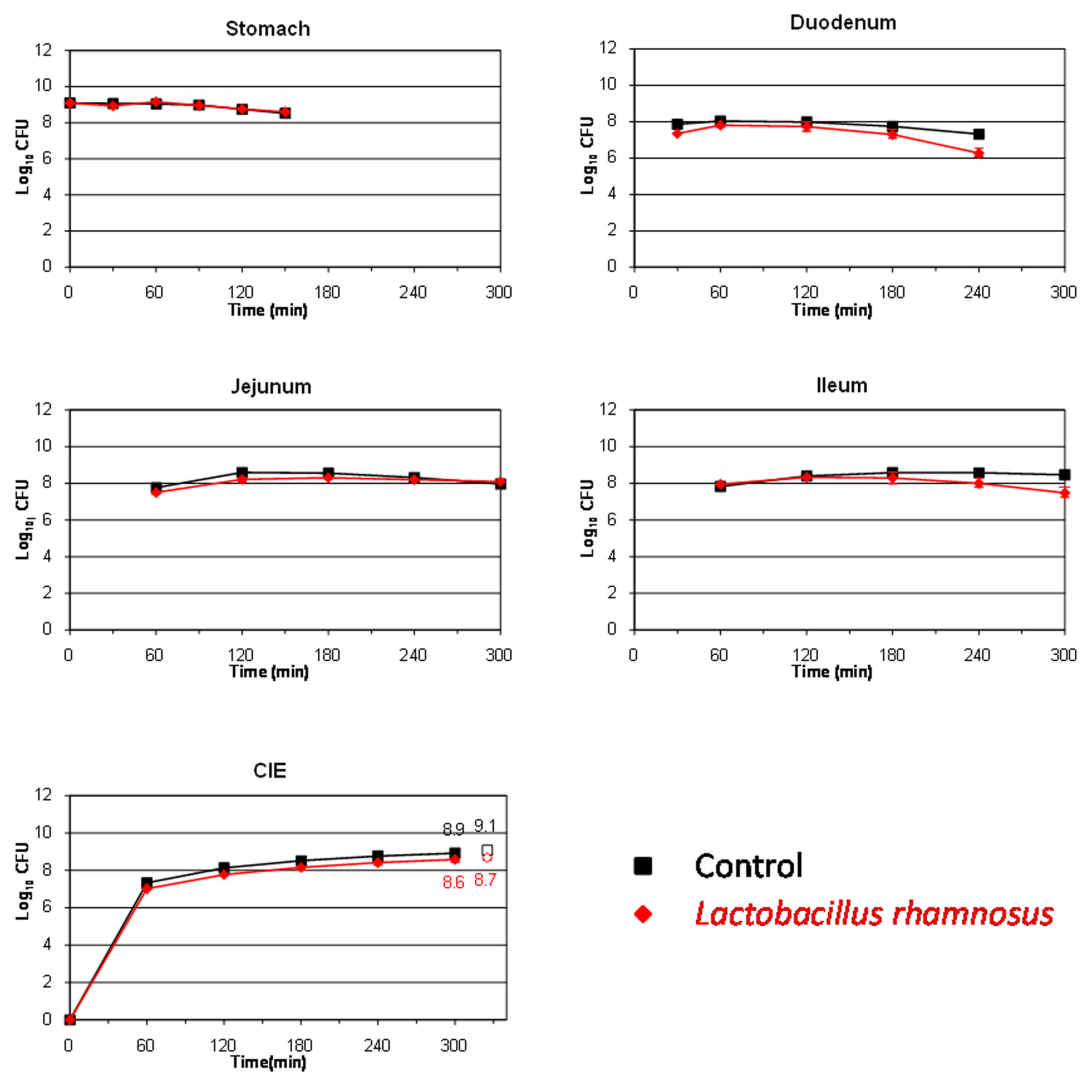
**Survival (plate counts versus time) of the probiotic strain *L. rhamnosus* GG in the different CIE of the TIM system compared to a non absorbable transit marker, blue dextran, used as control.** Results are expressed as log_10_ cfu ± SDs (*n* = 2).

To be effective and confer health benefits on the host, probiotics must be able to survive passage through the human stomach and small intestine and be present in sufficient number to colonize the colonic environment (Del Piano et al., 2006). The survival of the *L. pentosus* strains isolated from table olives was compared to that of the well-known probiotic *L. rhamnosus* GG (**Figure 4**). This microorganism was slightly affected during its transit through the stomach (only 0.1 log_10_ CFU reduction at time 150 min). Pitino et al. (2012) also found a high resistance of this species to stomach digestion in a dynamic gastric model. On the contrary, the population decreased in higher proportions at the end of its transit through the duodenum (∼1 log_10_ CFU at time 240 min) and it seemed to be not affected by the conditions occurring in the jejunum and ileum (no additional mortality was observed in either compartment). At 300 min, high levels for this LAB species were recovered from the ileal eﬄuents, decreasing by only 0.3 log_10_ CFU compared to the transit marker.

**Table 3** shows the percentage of survival obtained for the four assayed bacteria after their transit through the different compartments of the TIM system as well as in the ileal eﬄuents at the end of digestion. As can be clearly deduced even though *E. coli* O157:H7 is considered a highly acid resistant pathogen (Foster, 2004), this microorganism was the most affected bacteria by gastric digestion, with statistically significant differences (*p* < 0.05) compared to lactobacilli strains. *L. rhamnosus GG* was the most resistant bacteria during its transit through duodenum, although without significant differences according to a Fisher-LSD *post hoc* comparison test. This is in agreement with previous results obtained by Silva et al. (1987) and Pitino et al. (2012) who cataloged *L. rhamnosus* as an acid and bile-resistant species. Bile salts are described as toxic at high concentrations for bacterial cells by disorganizing the lipid bi-layer structure of the cellular membranes (Thanassi et al., 1997). This is presumably the reason why all assayed microorganisms reduced their population during their transit through the duodenum. On the contrary, the food-borne pathogen was the most adapted microorganism in the distal parts of the gastrointestinal tract (jejunum and ileum), with significant differences (*p* < 0.05) compared to the lactobacilli species in the ileum. Thereby, after gastric and small intestinal transit, the survival rate was statistically higher for *E. coli* O157:H7 EDL 933 (117.5%) than for the rest of bacteria (from 0.7 to 40.5%), indicating that a higher percentage of the pathogen population could enter into the colon compared to the lactobacilli species in the case of a hypothetical intake of contaminated olives. *L. pentosus* TOMC-LAB2 and TOMC-LAB4 have already shown promising probiotic characteristics (Bautista-Gallego et al., 2013) as well as a considerable capacity for adhesion to the olive epidermis (Arroyo-López et al., 2012). Population levels higher than 7.5 log_10_ CFU were recovered for both strains after their transit through the artificial ileum and before entering into the colon (survival percentages of 0.6 and 3.5% for *L. pentosus* TOMC-LAB2 and TOMC-LAB4, respectively), compared to 8.5 log_10_ CFU obtained for *L. rhamnosus* GG (survival percentage of 31.3%). This makes them good candidates for use as probiotic agents, which could increase the functional value of table olives.

**Table 3:** Survival (%) of the four tested strains at the end of *in vitro* digestions in the different compartments and ileal eﬄuents (CIE) of the TIM system, using fermented *Manzanilla* olives as food matrix.

Microorganism	E_150_	D_240_	J_300_	I_300_	CIE_300_	TF_325_
*E. coli* O157:H7 EDL 933	0.00 (0.00)^a^	0.01 (0.00)^a^	11.55 (8.69)^a^	45.27 (4.45)^a^	45.45 (11.66)^a^	117.47 (26.68)^a^
*L. rhamnosus* GG	32.80 (3.47)^c^	0.16 (0.05)^a^	2.52 (0.01)^a^	10.97 (4.55)^b^	31.29 (5.91)^a^	40.48 (7.37)^c^
*L. pentosus* LAB2	18.13 (0.05)^b^	0.001 (0.001)^a^	0.05 (0.00)^a^	0.24 (0.02)^c^	0.55 (0.13)^b^	0.74 (0.14)^b^
*L. pentosus* LAB4	24.11 (5.22)^b,c^	0.07 (0.05)^a^	0.47 (0.06)^a^	1.69 (0.66)^c^	3.47 (1.03)^b^	4.91 (1.04)^b,c^

*E_150_, D_240_, J_300_ and I_300_ stands for instantaneous percentages in the stomach (150 min), duodenum (240 min), jejunum (300 min), and ileum (300 min), respectively. CIE_300_ and TF_325_ stands for cumulative percentages from ileal eﬄuents (0-300 min) and cumulative percentages from ileal eﬄuents (0-300 min) plus final gastro-intestinal residue, respectively. SD in parentheses. Values followed by different superscript letters, within the same column, are significantly different (*p* < 0.05) according to a Fisher-LSD *post hoc* comparison test.*

## CONCLUSION

In summary, these results encourage further research on the development of table olives as a functional food, because, apparently, it is a good vehicle of microorganisms to the human body. Nevertheless, at the same time, they also show the necessity of good hygienic practices by olive manufacturers to avoid any possibility of intake of EHEC-contaminated olives, even though, apparently, the pathogen was not able to produce toxins during its transit through the upper human gastrointestinal tract following olive intake.

## Conflict of Interest Statement

The authors declare that the research was conducted in the absence of any commercial or financial relationships that could be construed as a potential conflict of interest.
